# Exploring the relationship between binge eating and differentiation of self: the mediating role of emotional distress and work stress

**DOI:** 10.3389/fnut.2024.1368995

**Published:** 2024-07-08

**Authors:** Ora Peleg, Meirav Idan, Ruth Katz

**Affiliations:** ^1^Max Stern Academic College of Emek Yezreel, Emek Yezreel, Israel; ^2^Department of Human Services, University of Haifa, Haifa, Israel

**Keywords:** binge eating, differentiation of self, emotional distress, work stress, anxiety, gender differences

## Abstract

Binge Eating Disorder (BED) is a prevalent eating disorder outlined in the DSM-5. Emotional distress (including stress, anxiety, and depression) stands out as a critical risk factor for developing eating disorders, and specifically BED. Recent studies have identified differentiation of self- a family pattern involving the ability to balance emotions and cognitions, as well as intimacy and autonomy—as a factor that exacerbates emotional distress. This relationship highlights the importance of addressing both emotional distress and family dynamics in understanding BED. While associations have been found between work-related factors and family dynamics with emotional distress, there has been limited investigation into the specific risk factors that are uniquely linked to BED. It was hypothesized that differentiation of self would relate to BED symptoms through the mediation of emotional distress and work stress. A systematic sampling method was applied to select a total of 275 participants for this study, with 60% women and 40% men (aged 20–45, *M* = 32.71, *SD* = 7.50). The findings suggest that low differentiation of self may increase vulnerability to BED symptoms by increasing susceptibility to emotional distress, including stress in the workplace. In addition, the analyses indicated that women reported higher levels of BED symptoms, while men reported higher levels of differentiation of self. The study sheds light on the contribution of unregulated family and emotional patterns to BED, providing valuable insights for organizations seeking to promote healthier work environments.

## Introduction

The prevalence of eating disorders is steadily increasing among women and men worldwide (1, 2). Among the various eating disorders outlined in the DSM-5 (3), binge eating disorder (BED) is particularly common, with a higher prevalence than anorexia nervosa and bulimia nervosa (4). BED has significant consequences for physical health (5, 6), and is often displayed with mental health disorders, such as anxiety, depression, and suicidal tendencies (7–9).

Research has found emotional distress to be a foremost catalyst of eating disorders (1, 2, 10, 11), particularly with regard to BED. There is also evidence that family dynamics play a crucial role in exacerbating, or conversely alleviating, emotional distress. An essential family pattern associated with both mental and physical health is differentiation of self (DoS). This family pattern entails balancing intimacy and autonomy in interpersonal relationships with significant others. Moreover, within the intrapersonal realm, it involves managing cognitions and emotions during stressful situations (1, 2). A low level of DoS has been associated with emotional distress (1, 2), depressive symptoms (12), somatic symptoms (13), an increased risk of eating disorders (1, 2), work stress, and workplace dysfunction, including diminished job satisfaction (14) and heightened burnout (15).

Despite the extensive evidence, theoretical and empirical literature on the symptoms of BED remains limited (4). Furthermore, only a small number of studies have investigated the factors that may contribute to its escalation (16, 17). Hence, the primary aim of the current study was to elucidate the intricate interplay between these dimensions and to explore whether a low level of DoS would contribute to the amplification of emotional distress and work stress, contributing in turn to the intensification of BED symptoms. In addition to this, considering the gender differences reported in studies on DoS, emotional distress, and, notably, some studies on BED, further investigation is imperative. Hence, another goal is to explore gender differences to ascertain whether, akin to other eating disorders, BED exhibits a higher prevalence among women. Additionally, examining gender differences aims to discern variations in the relationships among the research variables and BED. Exploring variations in the relationships between BED and associated risk factors, such as DoS and emotional distress, is crucial for several reasons. Firstly, understanding how these factors interact differently in individuals with BED can provide insights into the underlying mechanisms and pathways contributing to the development and maintenance of the disorder. Secondly, identifying specific patterns of association between BED and risk factors across genders can help tailor interventions more effectively, considering the unique needs and vulnerabilities of men and women.

### BED symptoms

Individuals with BED engage in episodes of extreme food consumption, eating significantly larger amounts of food than the average person under similar circumstances. These episodes involve rapid and secretive eating, a lack of hunger, and a perceived loss of control (18). Feelings of self-disgust, depression, and discomfort due to excessive fullness are common during these episodes (17). BED is not associated with compensatory behaviors like vomiting or excessive exercise (3), though efforts are made to maintain weight and diet (19). Individuals diagnosed with BED are often prone to various health problems, including high cholesterol levels, high blood pressure, and type 2 diabetes. These conditions further elevate the risk of more serious diseases, such as heart disease and stroke (6, 20).

BED has been recognized as a distinct eating disorder in the DSM-5 (3) and is now acknowledged as one of the most prevalent ones. It is estimated to occur among 3.5% of women and 2.0% of men (19), compared to 0.9 and 0.3%, respectively, for anorexia nervosa and 1.5 and 0.5%, respectively, for bulimia nervosa (16). BED typically develops during emerging adulthood, specifically between ages 18 and 26, whereas anorexia nervosa often manifests at an earlier age (18). Indeed, BED is particularly prevalent among young adults (5, 21).

BED is a complex condition influenced by genetic, environmental, and psychological factors. Studies have linked BED to such factors as BMI, metabolic issues, and disrupted hunger and satiety mechanisms (22). Obesity has also been associated with BED (9, 23).

Recent research has highlighted the significant contribution of emotional factors to BED. Emotional distress is considered crucial to the development and persistence of BED (7, 24). Individuals with BED may be at increased risk of suicide attempts (8), and more than half (58%) the individuals diagnosed with BED seek therapy (6).

### Emotional distress

Emotional distress encompasses symptoms of depression, anxiety, and stress, reflecting a state of emotional suffering. It arises from perceived difficulties in meeting daily demands and coping with stressful factors, often resulting in chronic somatic symptoms (25–27). Emotional distress serves as a crucial indicator of various mental disorders, such as depression and anxiety (3). High emotional distress has been associated with weight-related stigmatization reactions (28), food addiction (29), and excessive preoccupation with weight demonstrated through dietary restraint and dieting behaviors (30, 31). It has also been shown to be a primary risk factor for eating disorders, with individuals who experience stress, anxiety, and depression being particularly susceptible. Those exhibiting elevated levels of disordered eating attitudes and behaviors often display a heightened avoidance of emotions, and increased sensitivity. Consequently, adolescents and adults grappling with anxiety or depression may develop problematic or pathological eating patterns and thoughts (1, 2). Additionally, individuals with eating disorders and a history of childhood maltreatment exhibit heightened emotional overwhelming and increased post-stress body dissatisfaction, indicating altered emotional responses to stressors [e.g., (32)].

There is evidence of gender differences in emotional distress: elevated levels of persistent stress tend to be more prevalent among women (22, 24). Moreover, the work environment, including factors such as high workloads and time pressure, has been associated with elevated levels of emotional distress among employees, leading to various emotional responses, such as anxiety, depression, irritability, and burnout (33, 34).

### Work stress

Work stress has become increasingly prevalent in recent decades due to global, economic, and technological changes, as well as population growth and lifestyle modifications (35). It intensifies when there is a disparity between employees’ skills and the demands of their jobs (36) leading to difficulties in coping with work-related tasks (37, 38). Over 50% of workers consider work-related stress as the primary factor influencing their job, family life, and overall well-being, given the significant time spent at work (35). Studies on work stress have shown that both women and men experience increased work stress and reduced satisfaction with family and overall life when faced with long work hours and limited family time (33, 39).

Work stress has detrimental effects on employees, including diminished self-esteem (38), compromised job security, and reduced social relationships with colleagues (40). In addition, work stress has been found to be correlated with less satisfaction with work and in personal life (41), as well as increased anxiety and depression (33). Physiologically, work stress has been associated with imbalanced high-calorie eating habits, often perceived as comforting but leading to weight gain (42), increased body mass (BMI), and risk of BED (43). Furthermore, work stress has been identified as a risk factor for heart disease and cancer (35).

Work-related stress has been attributed to various factors, such as high workloads, long hours, excessive job demands (35, 37), and work–life imbalance (e.g., limited self-care or leisure time, fatigue) (39, 44). Moreover, unregulated family patterns may act as a risk factor: a low level of DoS can contribute to elevated stress levels and reduced job satisfaction among employees in organizations (44). Individuals with low DoS are indeed more prone to heightened work-related stress and may encounter greater challenges in managing stress within work environments (14).

### Differentiation of self

Family systems theory (45, 46) highlights the influence of emotional dynamics within the nuclear family on individuals’ self-perception and development. A key pattern in this theory is Differentiation of Self (DoS), which defines family members’ levels of emotional maturity, and is passed down from one generation to the next. DoS reflects emotional maturity and a strong sense of identity. Kerr and Bowen (46) distinguished between two realms of DoS, the intrapersonal and the interpersonal. At the intrapersonal level, it involves maintaining a healthy balance between emotions and rational thinking and expresses the individual’s ability to separate their instinctually driven emotional reaction from their goal-directed functioning. On an interpersonal level, high DoS entails establishing a harmonious equilibrium between intimacy and autonomy in meaningful relationships (2, 47).

Kerr and Bowen (46) argued that DoS is critical for mature development and the attainment of psychological health. Higher DoS allows one to experience strong affect or shift to calm, logical reasoning when circumstances dictate. Well differentiated individuals operate equally well on both emotional and rational levels while maintaining a measure of autonomy within their intimate relationships. In contrast, poorly differentiated persons tend to be more emotionally reactive (46) (p. 320), finding it difficult to remain relaxed when dealing with stressful situations. With intellect and emotions fused, they tend to make decisions based on what “feels right”; in short, they are trapped in an emotional world (45). The concept of DoS has been used to describe the way family patterns affect the trajectory of individual health and influence the extent to which individuals are able to take personal responsibility for age-appropriate tasks, and experience strong connections with significant others (48).

DoS encompasses four dimensions. Emotional reactivity relates to the intensity of emotions experienced and expressed in challenging circumstances. I-position reflects an individual’s ability to assert their needs, thoughts, and emotions while maintaining a sense of self without excessive reliance on others for validation. Emotional cutoff involves emotional and behavioral disconnections arising from difficulties in direct communication during challenging situations. Finally, fusion with others refers to the tendency to form dependent relationships characterized by blurred boundaries (49, 50).

From a gender perspective, men tend to report higher levels of emotional cutoff, while women tend to report higher levels of emotional reactivity and fusion with others (13, 50). Well-differentiated individuals tend to exhibit better coping abilities in stressful situations, experience greater well-being, possess a positive self-concept, and align their lives with their own desires (50). Conversely, poorly differentiated people are more likely to report higher levels of anxiety (51), stress, and depression (2), and to be at higher risk for type 2 diabetes mellitus (52) and eating disorders (10, 53).

DoS, shaped by interactions within the family of origin, can impact relationships at work, where individuals spend a substantial amount of time. Limited research suggests that individuals with lower levels of DoS are more likely to experience lower job satisfaction and conflicts in the workplace. These challenges may stem from difficulties in adapting to job demands, regulating emotions effectively, and relying heavily on others for emotional support (14, 46). As far as we know, no studies have examined the contribution of DoS and work stress to BED.

### Rationale and hypotheses

The literature highlights associations between BED symptoms, emotional distress (22), and work stress (43); between DoS and work stress (14); and between DoS and the risk of developing eating disorders (10). Yet, there is a lack of studies examining the combined contribution of these factors to the risk of BED among young adults, despite its high prevalence in this age group (18).

The goal of this study is to provide a framework that allows for an in-depth investigation of the complex relationships between BED symptoms, DoS (emotional reactivity, I-position, emotional cutoff, fusion with others) emotional distress (stress, anxiety, depression), and work stress, considering the potential mediating role of the latter two. Specifically, we examine how low DoS can heighten emotional distress and work stress, which in turn may exacerbate BED symptoms. This intricate interplay suggests that individuals with lower DoS are more vulnerable to stressors from work and family environments, which intensify emotional distress and may increase the risk of developing BED.

By incorporating all these variables and examining their associations, our research aims to provide a more nuanced understanding of the intricate pathway through which DoS, emotional distress, and work stress contribute to the development of BED. Moreover, we account for gender differences, acknowledging the observed distinctions in eating disorders (2) and specific symptoms of BED (7). Hence, we also investigate whether, in line with observations among young adults, BED tends to be more prevalent among women. This inquiry aims to deepen our comprehension of the risk factors associated with BED.

**Figure 1:**
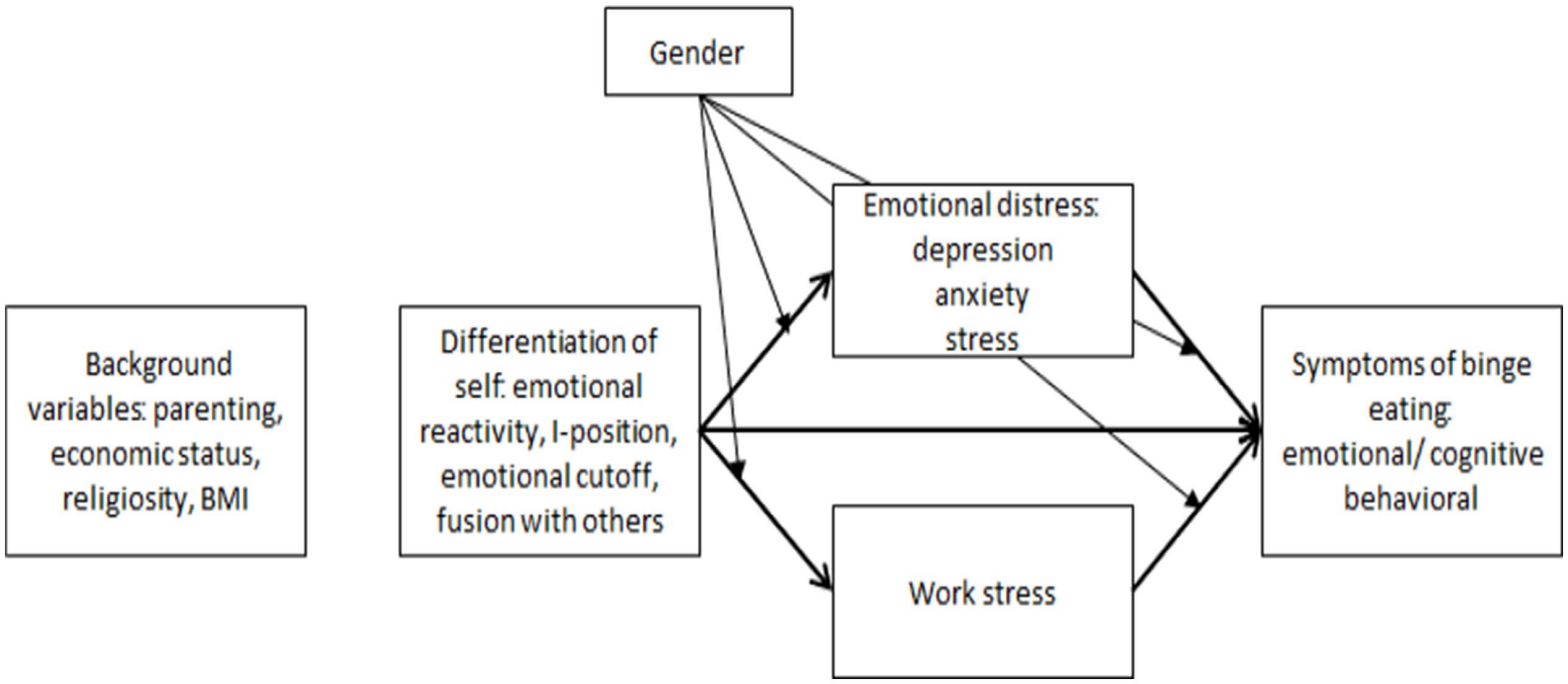
The theoretical model.

Accordingly, our research hypotheses were:

1. DoS (emotional reactivity, I-position, emotional cutoff, fusion with others) will be associated with BED symptoms (emotional/cognitive and behavioral), through the mediation of emotional distress (depression, anxiety, and stress) and work stress (Figure 1):
  - Elevated levels of emotional reactivity, emotional cutoff, and fusion with others are expected to be associated with higher emotional distress and work stress, and consequently with heightened symptoms of BED.
  - Conversely, a high level of I-position is anticipated to mitigate emotional distress and work stress, thereby being associated with a reduced likelihood of experiencing BED symptoms.
2. Gender differences are expected: women will be more likely to report higher levels of BED symptoms, emotional reactivity, fusion with others, and emotional distress than men, while men will be more likely to report higher levels of emotional cutoff than women.
3. Gender will moderate the associations between DoS and emotional distress and work stress, as well as between these study variables and BED symptoms. Specifically, these associations will be stronger for women than for men.

## Methods

### Sample

A systematic sampling method was applied, and a representative sample of the general Hebrew-speaking population of Israel was recruited. The sample includes a total of 275 Israeli participants, with 60% women and 40% men aged 20–45 (*M* = 32.71, *SD* = 7.50). Inclusion criteria required participants to be working individuals who were fluent in Hebrew, capable of understanding the questionnaire, and providing responses to the survey.

### Instruments

The ***Binge Eating Scale*** (BES) (54) was used to assess the severity of BED symptoms. For the purpose of this research, the questionnaire was translated into Hebrew by an expert and then back-translated to English by another expert. The second author checked the congruency between the versions, and the final versions were reviewed by the three authors of the study. The questionnaire consists of 16 items divided into two scales: emotional/cognitive (sample item: “I do not feel self-conscious about my weight or body size when I’m with others”), and behavioral. Each item is rated on a Likert scale, with scores ranging from 0 to 2 or 0 to 4 (54). The scoring range for the questionnaire is 0–46, where 0–17 = no evidence of BED symptoms; 18–26 = a moderate degree; and 27 or higher = a severe degree. The cutoff point defining the presence of BED symptoms is set at 17 (55–58). The BED questionnaire demonstrated good internal consistency for the total score (α = 0.84), and the feelings and thoughts scale (α = 0.81), but low internal consistency for the behavior scale (α = 0.62). Due to this latter low internal consistency and the high correlation between the two subscales, the total score was used in the present study.

The ***Short Depression*, *Anxiety and Stress Scale*** (DASS–21) (59), translated to Hebrew (1), was used to assess emotional distress. This 21-statement self-report questionnaire assesses symptoms in three areas (divided into three subscales): depression, anxiety, and stress. Sample item for stress: “I find it difficult to relax.” Participants respond on a 4-point Likert scale (0 = strongly disagree, 3 = strongly agree). The questionnaire is suitable for both adults and youth aged 14 and above. In the current study, high internal consistency was found for the total score of the questionnaire (α = 0.94), and good internal consistencies were observed for the three subscales: depression (α = 0.88), anxiety (α = 0.82), and stress (α = 0.88).

The ***Job Stress Questionnaire*** (60), based on the Job-Related Tension Inventory (JRTI) (60), is a 15-item self-report tool used to assess employees’ perceptions of work stress. We used the Hebrew version in the current study (Hebrew). Sample item: “How often are you bothered at work by not knowing what exactly the people you work with expect from you?” Participants respond on a Likert scale ranging from 1 (never) to 5 (almost all the time). Scores are averaged to calculate an overall score, ranging from 1 to 5. The questionnaire has demonstrated good internal consistency (α = 0.92).

**The *Differentiation of Self Inventory-Revised*** (DSI-R) (48, 61) is a self-report questionnaire that has been translated to Hebrew by Peleg (62, 63). It assesses an individual’s level of DoS and their relationships including with their family of origin. The 46 items are divided into four subscales: emotional reactivity, I-position, emotional cutoff, and fusion with others. Sample item: “I have difficulty expressing my feelings toward people who are important to me” (emotional cutoff). Participants respond on a Likert scale ranging from 1 (not at all true for me) to 6 (very true for me). The total score of the questionnaire is calculated by averaging the raw score of all the items in each of the four scales, with a higher score in the I-position scale, along with lower scores in emotional reactivity, emotional cutoff, and fusion with others scales, indicating a higher level of DoS. The questionnaire has demonstrated high internal consistency in the present study: for the total score (α = 0.90), and for the four subscales, emotional reactivity (α = 0.89), I-position (α = 0.81), emotional cutoff (α = 0.82), and fusion with others (α = 0.81).

A ***demographic questionnaire*** was specifically developed for the present study. It included the following information: gender, age, marital status (married/in a relationship, single, divorced/widowed), parentage, number of children, education level, employment status (full-time salaried employee, part-time, self-employed), occupation, weekly work hours, economic status, religiosity (secular, traditional, religious, ultra-Orthodox), has a chronic illness, has learning disabilities, and weight and height (BMI). In assessing the economic status of participants, we used both a subjective assessment of their economic situation and an assessment based on the average income in Israel; the correlation between these two measures was *r* = 0.53 (*p* < 0.001). To provide a comprehensive representation of the participant’s economic situation, an average between these two assessments was calculated.

### Procedure

A survey company was contracted to assist with the distribution of the questionnaire to individuals who met the predetermined inclusion criteria using systematic sampling. The survey company uses a systematic sampling method (approacing every i^th^ person), using an on-line or direct communication with them. All participants were provided with a detailed explanation of the study’s purpose and procedures. Participants were assured that their details would be kept anonymous and that their responses would be treated with utmost discretion. They were also informed of their right to withdraw from the study at any point without facing any consequences. Upon understanding the study’s requirements and providing their voluntary consent, participants signed an informed consent form to signify their agreement to participate. They were then given access to the online questionnaire, which typically took 20–30 min to complete. Data collection took approximately 1 month. The study was approved by the University Ethics Committee (Approval No: 231/23).

### Data analysis

Data analysis was done with SPSS software, version 28. Means and standard deviations were computed for continuous background variables; frequencies and percentages were calculated for categorical background variables. Means and standard deviations were calculated for the study variables, and Pearson correlations were calculated to examine their associations. Internal consistencies of the research variables were assessed by Cronbach’s alpha (α). For BED symptoms and emotional distress, abnormality categories were determined based on the guidelines provided by the measurement tools. To explore the potential associations of background characteristics with the study variables, t-tests were performed comparing the study variables with dichotomous background variables (e.g., gender), and Pearson correlations were calculated between the study variables and the main continuous background variables (e.g., age). As the emotional distress variable and its dimensions were found to deviate from a normal distribution (skewness index = 1.09–1.68, SE = 0.15), a logarithmic transformation was applied to them. The two economic status variables (subjective assessment and assessment based on average income) were found to follow a normal distribution (skewness = −0.44, SE = 0.15, and skewness = −0.03, SE =0.15, respectively) and were thus treated as continuous variables.

The first hypothesis was evaluated through Pearson correlations between the independent (DoS), mediating (emotional distress, work stress), and dependent (BED symptoms) variables, as well as multiple linear regressions. For BED symptoms, background variables were entered in the first step, the independent variable (DoS) in the second step, and the mediating variables (emotional distress and work stress) in the third step. Regressions were also calculated for the mediating variables, with the background variables entered in the first step and the independent variable in the second.

To test the mediation model, structural equation modeling (SEM) was applied using AMOS software, version 28. The measurement model, which includes correlations between latent variables, was estimated first, followed by the mediation model. Fit indices were used to assess model fit, where a Cmin/df value of less than 3 was considered indicative of a good fit (64); NFI, NNFI, and CFI values greater than 0.90 represented a reasonable fit and values greater than 0.95 indicated a good fit (64, 65); and RMSEA values below 0.08 indicated a reasonable fit and values below 0.05 indicated a good fit (66). The mediation analysis was calculated using path analysis with 5,000 bootstrap samples and a bias-corrected confidence interval of 95%. Continuous variables were standardized. Control variables were gender, parentage, economic status, level of religiosity, and BMI.

The second research hypothesis, which focused on gender differences in the research variables, was examined with multivariate analyses of variance (MANCOVA), controlling for parentage, economic status, level of religiosity, and BMI.

## Results

### Descriptive findings

The distribution of the background variables is shown in Table 1. Approximately 10% of the study participants reported having chronic illnesses. The average body mass index (BMI) of participants was around 25, and about 56% of the participants reported normal weight or underweight.

**Table 1:** Distribution of background variables (*N* = 275).

Variable		Values
Gender, n (%)	Male	110 (40.0%)
	Female	165 (60.0%)
Age, years M(SD)	Range: 20–45 years	32.71 (7.50)
Marital status, n (%)	Married, in a relationship	197 (71.7%)
	Single	71 (25.8%)
	Divorced/widowed	7 (2.5%)
Children, n (%)	Yes	161 (58.5%)
Number of children, M(SD)	Range: 1–9	2.48 (1.31)
Education level, n (%)	Less than high school	13 (4.7%)
	High school	48 (17.5%)
	Vocational	27 (9.8%)
	College student	34 (12.4%)
	Bachelor’s degree	104 (37.8%)
	Graduate degree	49 (17.8%)
Years of education, M(SD)	Range: 8–22 years	14.75 (2.57)
Employment status, n (%)	Full-time salaried employee	220 (80.0%)
	Part-time salaried employee	45 (16.4%)
	Self-employed	10 (3.6%)
Occupation, n (%)	Manager	42 (15.3%)
	Academician	43 (15.6%)
	Technician, agent	24 (8.7%)
	Clerical/office worker	61 (22.2%)
	Sales and services	20 (7.3%)
	Skilled/unskilled worker in agriculture or industry	25 (9.1%)
	Other	60 (21.8%)
Weekly work hours, M(SD)	Range: 7–90 h	40.32 (11.56)
Economic status (subjective assessment), n (%)	Very bad	7 (2.5%)
	Bad	17 (6.2%)
	Moderate	123 (44.7%)
	Good	110 (40.0%)
	Very good	18 (6.5%)
Income relative to national average, n (%)	Much below average	34 (12.4%)
	Below average	74 (26.9%)
	Average	86 (31.2%)
	Above average	69 (25.1%)
	Much above average	12 (4.4%)
Religiosity, n (%)	Secular	129 (46.9%)
	Traditional	56 (20.4%)
	Religious	54 (19.6%)
	Ultra-Orthodox	36 (13.1%)
Chronic illnesses, n (%)	Yes	26 (9.5%)
Learning disability, n (%)	Yes	52 (18.9%)
BMI, M(SD)	Range: 17–55	25.46 (5.32)
BMI categories, n (%)	Underweight	11 (4.0%)
	Normal	143 (52.0%)
	Overweight	83 (30.2%)
	Obese	38 (13.8%)

Table 2 presents the distribution of the study variables and Pearson correlations between them. Significant moderate correlations were found between most of the variables. The three dimensions of DoS that point to low differentiation (namely, emotional reactivity, emotional cutoff, and fusion with others) were positively correlated with BED symptoms, emotional distress, and work stress. Conversely, I-position and the total score of DoS (both of which indicate high differentiation) were negatively correlated with these same study variables. BED symptoms, emotional distress, and work stress were all positively correlated.

**Table 2:** Distribution of the study variables and Pearson correlations between them (*N* = 275).

	M(SD)	1.	2.	3.	4.	5.	6.	7.	8.	9.	10.	11.
1. BED symptoms	9.37 (6.70)	1										
2. Emotional distress: total	21.68 (20.24)	0.40	1									
3. Depression	6.63 (7.36)	0.40	0.87	1								
4. Anxiety	5.37 (6.45)	0.31	0.80	0.68	1							
5. Stress	9.69 (8.42)	0.36	0.92	0.73	0.65	1						
6. Work stress	2.10 (0.67)	0.34	0.48	0.47	0.44	0.47	1					
7.DoS: total	3.86 (0.62)	−0.33	−0.61	−0.58	−0.48	−0.63	−0.53	1				
8. Emotional reactivity	3.39 (1.05)	0.29	0.57	0.50	0.47	0.63	0.46	−0.88	1			
9. I-position	4.07 (0.80)	−0.19*	−0.24	−0.30	−0.20*	−0.24	−0.25	0.45	−0.18*	1		
10. Emotional cutoff	2.58 (0.85)	0.18*	0.42	0.41	0.34	0.41	0.37	−0.67	0.43	–.11^ns^	1	
11. Fusion with others	3.67 (0.81)	0.26	0.43	0.40	0.31	0.46	0.39	−0.77	0.75	–.07^ns^	0.29	1

All r significant at *p* < 0.001, or *p < 0.01, and ns- non- significant.

The categorization of BED scores revealed that approximately 88% of participants were classified as having no evidence of BED (a score of 0–17), while about 12% were classified as having a moderate (18–26) or severe (27+) degree of the disorder. In terms of gender differences, 21 women (12.7%) and 12 men (10.9%) were classified as suffering from moderate or severe BED, with no significant gender difference (*Z* = 0.45, *p* = 0.649). Similarly, the scores for the emotional distress dimension were also categorized.

### Relationships between background and research variables

We examined relations between background and study variables to identify potential confounding factors that should be controlled for when analyzing the research hypotheses.

For BED symptoms, a significant gender difference was observed, with women reporting higher levels (*M* = 10.13, *SD* = 6.68) than men (*M* = 8.23, *SD* = 6.59) [*t*(273) = 2.32, *p* = 0.021]. Furthermore, there was a positive correlation between BMI and BED symptoms: the higher the BMI, the higher the level of BED symptoms (*r* = 0.27, *p* < 0.001).

With respect to emotional distress, significant differences in the total score were observed with regard to the parenting variable: non-parents reported higher levels of emotional distress (*M* = 28.00, *SD* = 22.90) than parents (*M* = 17.20, *SD* = 16.81) [*t*(262.13) = 4.67, *p* < 0.001]. Significant differences in the total score and all three dimensions (depression, anxiety, and stress) were also found for economic status: the better the financial situation, the lower the emotional distress (*r* = −0.14, *p* = 0.026). Significant differences in the total score were also found for degree of religiosity: traditional, and secular participants reported higher levels of emotional distress (*M* = 24.69, *SD* = 21.55) than religious and ultra-orthodox participants (*M* = 15.49, *SD* = 15.58) [*t*(273) = 3.61, *p* < 0.001]. Findings for the three dimensions were similar. In addition, a positive correlation was found between BMI and emotional distress (total score and all dimensions), indicating that the higher the BMI, the greater the emotional distress (*r* = 0.19, *p* = 0.001).

Finally, the total DoS score was found to be higher among men (*M* = 4.04, *SD* = 0.57) than women (*M* = 3.74, *SD* = 0.62), pointing to gender differences [*t*(273) = 4.18, *p* < 0.001]. Moreover, parents reported higher DoS (*M* = 3.94, S*D* = 0.60) than non-parents (*M* = 3.75, *SD* = 0.62) [*t*(273) = 2.55, *p* = 0.011]. Lastly, a positive correlation was found between participants’ economic status and DoS: the better the financial situation, the higher the DoS (*r* = 0.14, *p* = 0.018). In light of these findings, the research hypotheses were examined controlling for gender, parentage, economic status, level of religiosity, and BMI. No significant relationships or differences were found between work stress and the background variables examined in the study.

### Examination of research hypotheses

#### Relationships between the study variables

To further examine these relationships, multiple regression analyses were calculated controlling for gender, parentage, economic status, level of religiosity, and BMI. Owing to a high correlation between two dimensions of DoS, namely, emotional reactivity and fusion with others (*r* = 0.75), as well as between the three dimensions of emotional distress (*r* = 0.65 to *r* = 0.73), total scores were used for DoS and emotional distress to avoid collinearity. Regarding BED, in the first step the background variables were entered, followed by the total score of DoS in the second step, and finally the total scores of emotional distress and work stress in the third step.

The regression model for BED symptoms yielded significant results, explaining 23% of the variance. In the first step, 10% of the variance was significantly accounted for by the background variables, so that for women, and for higher BMI level, there were higher levels of BED symptoms. The addition of DoS in step 2 was significant, adding 8% to the explained variance (the lower the level of total DoS, the higher the level of BED symptoms). Levels of emotional distress and work stress in step 3 added 5% to the explained variance: the higher these levels, the higher the level of BED symptoms.

With respect to emotional distress (total and the three dimensions) and work stress, all five regression models were significant, explaining 28–44% of the variance in these two variables. Across all models, the inclusion of DoS in step 2 significantly increased the explained variance by 18–35%. Being a parent, level of religiosity, and BMI were identified as significant predictors of emotional distress. Moreover, the addition of the total DoS score was significant, suggesting that the lower the level of DoS, the higher the level of emotional distress. Similar findings were found for the dimensions of depression, anxiety, and stress. After controlling for background variables, negative associations were found between DoS and all three dimensions of emotional distress. With respect to work stress, a significant association was found with DoS: the lower the DoS, the higher the level of work stress.

#### Hypothesis 1: The mediation model

According to the first hypothesis, emotional distress and work stress will mediate the relationship between DoS and BED symptoms (Figure 1). Due to the abovementioned high correlation between emotional reactivity and fusion with others and between the three dimensions of emotional distress, the hypothesis was tested by structural equation modeling (SEM), with gender, parentage, economic status, level of religiosity, and BMI defined as control variables. DoS was defined as the independent variable (latent variable) when the four dimensions were defined in the positive direction (a higher score representing a better result). Emotional distress and work stress were defined as the mediating variables (latent variables), while the level of BED symptoms (emotional/cognitive and behavioral) was defined as the dependent variable (latent variable with both dimensions).

**Figure 2:**
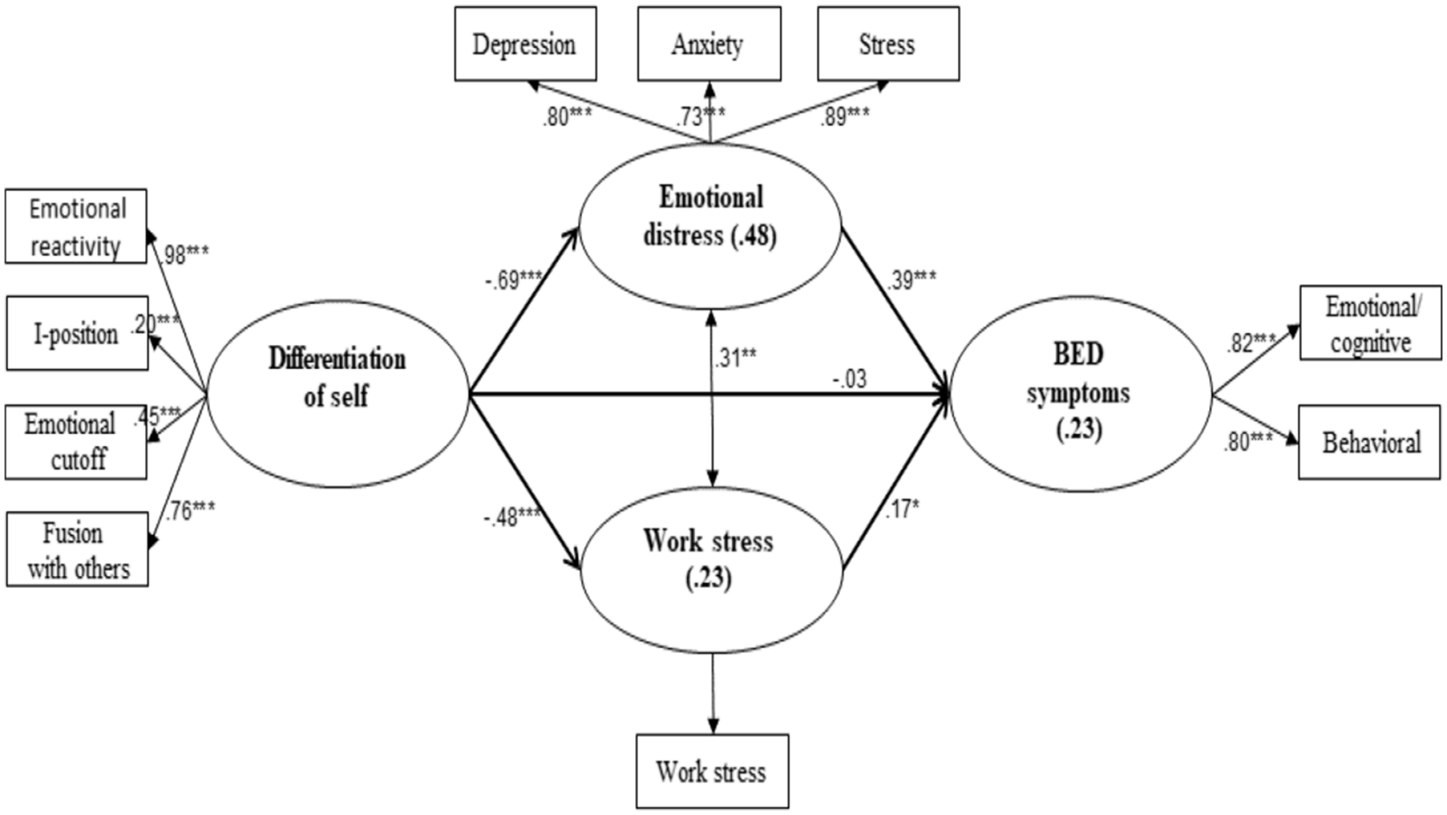
Structural equation modeling for emotional distress and work stress as mediating the association between Dos and BED symptoms. *p < 0.05, **p < 0.01, ***p < 0.001. Observed variables are inside rectangles; latent variables, together with the percent of explained values (*R*^2^), are in ellipses. Values next to unidirectional arrows are β values; the value next to the bidirectional arrow is a Pearson’s r. Control variables are excluded for purposes of clarity.

Examining the measurement model, results indicated a good fit to the data: Cmin/df = 2.292, NFI = 0.951, NNFI = 0.951, CFI = 0.971, RMSEA = 0.069. These fit indices suggest that the measurement model adequately represents the relationships between the observed indicators and the latent variables. Results for the mediation model also indicated a good fit: Cmin/df = 1.660, NFI = 0.935, NNFI = 0.959, CFI = 0.973, RMSEA = 0.049. This latter model is shown in Figure 2.

Results yielded significant associations between the research variables, such that the lower the level of DoS, the higher the levels of emotional distress and work stress; in turn, the greater the emotional distress and work stress, the higher the level of BED symptoms. Indeed, according to these direct relationships, the overall mediation effect was found to be significant: effect = −5.53, *SE* = 3.10, *p* < 0.001, 95%CI = −14.45, −2.89. The two specific effects were also significant; for the relationship of DoS → emotional distress→BED symptoms: effect = −0.91, *SE* = 0.30, *p* < 0.001, 95%CI = −1.57, −0.39; for DoS → work stress→BED symptoms: effect = −0.28, *SE* = 0.13, *p* = 0.019, 95%CI = −0.55, −0.04. These values suggest that the indirect pathways through emotional distress and work stress explain a significant portion of the relationship between DoS and BED symptoms, supporting the first research hypothesis.

#### Hypotheses 2 and 3: Gender differences and moderation effects

The second hypothesis suggested that women will report higher levels of emotional reactivity, fusion with others, emotional distress, and BED symptoms, while men will report higher levels of emotional cutoff. Multivariate analyses of variance (MANCOVA), controlling for parentage, economic status, level of religiosity, and BMI, indicated that women reported higher levels of BED symptoms and fusion with others, while men reported higher levels of DoS (total score; Table 3). Together, these findings partially support the second hypothesis.

**Table 3:** Means, standard deviations, and f values for the study variables by gender (*n* = 275).

	Total M(SD) (skewness, kurtosis)	Females M(SD)	Males M(SD)	*F*(1, 269), (p) (η^2^)
BED symptoms	9.37 (6.70) (0.86, 0.64)	10.13 (6.68)	8.23 (6.59)	7.63 (*p* = 0.006) (η^2^ = 0.028)
Emotional distress: total	21.68 (20.24) (1.44, 2.69)	22.55 (20.00)	20.38 (20.60)	1.59 (*p* = 0.208) (η^2^ = 0.006)
Depression	6.63 (7.36) (1.68, 3.67)	6.70 (7.38)	6.51 (7.37)	0.21 (*p* = 0.646) (η^2^ = 0.001)
Anxiety	5.37 (6.45) (1.53, 1.98)	5.53 (6.34)	5.13 (6.63)	1.25 (*p* = 0.264) (η^2^ = 0.005)
Stress	9.69 (8.42) (1.09, 1.40)	10.32 (8.39)	8.75 (8.42)	3.21 (*p* = 0.074) (η^2^ = 0.012)
Work stress	2.10 (0.67) (0.41, −0.25)	2.12 (0.68)	2.07 (0.66)	0.23 (*p* = 0.631) (η^2^ = 0.001)
DoS: total	3.86 (0.62) (−0.28, 0.63)	3.74 (0.62)	4.04 (0.57)	15.64 (*p* < 0.001) (η^2^ = 0.055)
Emotional reactivity	3.39 (1.05) (0.09, −0.38)	3.66 (1.03)	2.98 (0.94)	1.25 (*p* = 0.264) (η^2^ = 0.005)
I-position	4.07 (0.80) (−0.22, 0.17)	4.03 (0.82)	4.12 (0.76)	0.09 (*p* = 0.765) (η^2^ = 0.001)
Emotional cutoff	2.58 (0.85) (0.49, 0.42)	2.59 (0.88)	2.56 (0.81)	0.08 (*p* = 0.774) (η^2^ = 0.001)
Fusion with others	3.67 (0.81) (−0.32, 0.27)	3.84 (0.79)	3.40 (0.77)	20.21 (*p* < 0.001) (η^2^ = 0.070)

For skewness values: SE = 0.15, for Kurtosis values: SE = 0.29. For females: skewness values = −0.50 to 1.74, SE = 0.19; kurtosis values = −0.27 to 3.67, SE = 0.38. For males: skewness values = −0.22 to 1.61, SE = 0.23; kurtosis values = −0.57 to 3.90, SE = 0.46.

In addition to examining direct gender differences, its moderating effect on the model associations was examined. As shown in Table 2, the correlation between two aspects of DoS (emotional reactivity and fusion with others) was *r* = 0.75 (*p* < 0.001), thus leading to collinearity in any model involving the DoS aspects separately (VIF = 3.23). Thus, the moderating effects were examined regarding the total DoS score. All moderating effects for gender were found to be non-significant: for the associations between DoS and emotional distress (*β* = 0.01, *p* = 0.867), DoS and work stress (*β* = −0.02, *p* = 0.786), DoS and BED symptoms (*β* = 0.11, *p* = 0.220), emotional distress and BED symptoms (*β* = 0.03, *p* = 0.709), and work stress and BED symptoms (*β* = 0.11, *p* = 0.172). Thus, despite significant gender differences observed in DoS and BED symptoms, the model associations were not moderated by gender, refuting the third hypothesis.

## Discussion

The main study objective was to investigate the mechanism that activates BED symptoms, and the pathway through which family, work, and personality factors contribute to their development. Taken together, the findings suggest that low DoS (the predictor) may increase vulnerability to BED symptoms (the outcome) by increasing susceptibility to emotional distress, including stress in the workplace (mediators). In addition, the analyses pointed to certain gender differences: women reported higher levels of BED symptoms, while men reported higher levels of DoS.

### Emotional distress and work stress mediate the relationship between DoS and the risk of BED symptoms

A major tenet of the current study was that low DoS might increase the likelihood of BED symptoms by increasing vulnerability to emotional distress and work stress. Indeed, the findings revealed that the severity of emotional distress and work stress mediated the relationship between BED and DoS. These results align with previous research that has linked DoS to emotional well-being and mental health outcomes. They reinforce the notion that emotional maturity and the ability to maintain a balance between emotional and intellectual functioning are crucial for psychological well-being in various domains of life (49, 50).

Apparently, well-differentiated individuals are more likely to navigate uncertain circumstances and emotionally charged events using calm, rational thinking (67). In contrast, poorly differentiated individuals may find it difficult to maintain a clear sense of self and have difficulty adhering to their personal convictions instead of conforming to others’ expectations. They also may tend to isolate themselves from others and their emotions when faced with intense interpersonal experiences, or conversely, create dependent relationships and lean on close people, finding it difficult to maintain healthy boundaries in relationships (51, 68). While these maladaptive coping patterns may provide temporary relief, in the long term they can increase emotional burden, a sense of being overwhelmed, which may further exacerbate emotional distress and consequently elevate the risk of BED. It is suggested that the risk of BED increases because individuals may use BED as a way to alleviate negative emotions (69). Furthermore, it is likely that emotional distress can disrupt normal appetite regulation mechanisms, leading to dysregulated eating patterns (70). It is possible that difficulties regulating their emotions and their relationships with significant others leads such individuals to eat in an effort to numb their emotions and maintain a sense of control (2, 69), turning to food as a mechanism for coping with emotional distress. Such reliance on food can hinder the use of healthier skills that can manage emotions effectively.

The current results indicate that not only personal distress, but also stress in the work environment, mediates the relationship between DoS and BED. The workplace can be seen as a social and emotional system that encompasses interpersonal interactions, rules, expectations, and roles. This environment acts as a platform where individuals express what they have learned from their family of origin as they mature (44, 45). Research suggests that even individuals who are well-differentiated may experience increased stress situations; however, they respond and react to these situations differently than poorly differentiated individuals (71). The present results thus partially support findings indicating that low DoS predicts high levels of work stress and low job satisfaction (14), as well as higher levels of conflicts (14). Moreover, poorly differentiated individuals tend to have decreased enthusiasm and increased stress and burnout (15), as well as a high dependency on others (25, 35). Another potential explanation is that poorly differentiated individuals may struggle to seek support in stressful situations, including the workplace (15, 71). As a result, they may find it challenging to cope with work-related stress and navigate workplace dynamics effectively (36). This can lead to decreased job satisfaction and increased conflicts in their professional lives. It is possible that the difficulty in regulating emotions in the workplace channels the emotional distress into unregulated eating patterns, and that increased work stress and burnout can lead to decreased awareness of eating, decreased intuitive eating, and a sense of lack of control overeating (31, 43, 72).

#### Gender differences

Consistent with prior research (6, 22), men in our study exhibited lower scores on BED symptoms than women. Women may be more influenced by societal pressure surrounding the ideal of thinness and beauty (2), potentially making them more susceptible to symptoms associated with BED. However, it is important to note the small difference in percentages between women and men diagnosed with moderate or severe BED in the current sample (12.7% vs. 10.9%, respectively). This emphasizes the significance of considering the experiences and challenges faced by both genders in relation to BED. In contrast, men exhibited higher DoS, suggesting a greater inclination toward emotional separateness from others. These gender differences may arise from a range of factors, such as societal expectations, cultural norms, coping mechanisms, and stress responses (2, 21, 31, 73).

#### Limitations and future research

Our results should be interpreted with caution due to several study limitations. Firstly, the sample size is relatively small, which may impact the generalizability of the findings. Furthermore, the sample was composed predominantly of individuals from middle and upper-class backgrounds, limiting the applicability of the findings to people of lower socioeconomic backgrounds. To address these limitations, future research should include larger and more diverse samples, particularly in terms of socioeconomic groups. This would facilitate a more comprehensive understanding of the relationships under investigation and allow for more accurate generalizations to be made.

Secondly, it is important to acknowledge other variables that might serve as moderators in the relationships between DoS and emotional distress; DoS and work stress; and emotional distress, work stress, and BED. It would be worthwhile to explore such socioeconomic demographic variables as cultural/ethnic affiliation and level of education; such workplace variables as workload and income level; such childhood family variables as parenting styles and family atmosphere; and such personality variables as assertiveness, agreeableness, and psychological flexibility. Indeed, several studies investigating the impact of psychological flexibility on eating-related concerns have indicated a heightened risk of eating disorders (74) and emotional eating (75) among individuals with extreme obesity, who often exhibit elevated emotional distress and low psychological flexibility. Further investigation is warranted to explore the specific mechanisms underlying these relationships for a more comprehensive understanding of the phenomenon.

Thirdly, the current study was conducted in a cross-sectional setting, capturing data at a single point in time. This limits the ability to measure the development and progression of BED symptoms over time. It is recommended to conduct a longitudinal study, allowing for the assessment of the development and trajectory of BED symptoms among young people over an extended period.

#### Theoretical conclusions and contributions

Notwithstanding the study’s limitations, it offers significant contributions. From a theoretical perspective, previous research findings have shown that emotional distress mediated the relationship between DoS and the risk of eating disorders. The current study expands upon the existing literature by incorporating such novel variables as family, personality, and work-related factors in the investigation of BED. This enhances our understanding of the complex nature of BED beyond the traditional focus on personality measures (23, 24). Furthermore, the study sheds light on the contribution of unregulated family and emotional patterns to BED, providing valuable insights for organizations seeking to promote healthier work environments and improve employee well-being. The findings emphasize that unregulated family patterns and conflicts can add to stress levels and dissatisfaction, which can spill over into the workplace. Therefore, improving DoS within individuals can help organizations mitigate the negative effects of family dynamics on workplace satisfaction and stress levels.

#### Relevance for clinical practice

In practical terms, increased knowledge of BED may contribute to the development of effective prevention strategies. By identifying risk factors and early signs, interventions can be designed to intervene and prevent the onset of BED before it becomes chronic. The study findings can thus contribute to evidence-based interventions for BED, encouraging healthier eating patterns by focusing on emotional triggers in individuals’ lives, particularly within the family and the work environment. Organizations can support employees by addressing disordered eating symptoms, providing resources and interventions to improve overall well-being. This includes promoting healthy eating habits, offering counseling or employee assistance programs, and creating a supportive work environment that prioritizes work–life balance and stress management. By supporting individuals in the development of healthy coping mechanisms and interpersonal relationships, organizations can potentially reduce work stress and consequently the risk of BED. In addition, it is suggested that psychologists and family therapists who assist individuals suffering from Binge Eating Disorder (BED) should focus on improving DoS. This will lead to improved functioning both within their family and workplace environments.

Finally, from methodological and therapeutic perspectives, ongoing research can play a crucial role in refining the diagnostic criteria for BED, enabling better identification and monitoring of treatment outcomes. Advancements in understanding BED will pave the way for improved treatment outcomes.

In short, the research findings underline the importance of considering both personal and contextual factors in understanding, addressing, and treating BED. The study thus contributes to our understanding of the complex interplay between DoS, emotional distress, work stress, and BED.

## Data availability statement

The raw data supporting the conclusions of this article will be made available by the authors, without undue reservation.

## Ethics statement

The studies involving humans were approved by the Ethics Committee of the University of Haifa, within the Department of Human Services. The studies were conducted in accordance with the local legislation and institutional requirements. The participants provided their written informed consent to participate in this study.

## Author contributions

OP: Conceptualization, Data curation, Investigation, Methodology, Project administration, Software, Supervision, Validation, Visualization, Writing – original draft, Writing – review & editing. MI: Conceptualization, Data curation, Investigation, Project administration, Resources, Validation, Visualization, Writing – original draft, Writing – review & editing. RK: Conceptualization, Investigation, Project administration, Supervision, Validation, Visualization, Writing – review & editing.
